# Illinois Associations

**Published:** 1898-06

**Authors:** 


### ILLINOIS ASSOCIATIONS.

Illinois State Veterinary Medical Association. Officers: President, Albert Babb, Springfield; Vice-President, W. J. Martin, Kankakee; Treasurer, R. G. Walker, Chicago; Secretary, S. S. Baker, Chicago. Meeting, Chicago, at call of the President.

Chicago Veterinary Society. Officers: President, Robert G. Walker; First Vice-President, Lawrence Campbell; Second Vice-President, A. E. Flowers; Third Vice-President, A. Worms; Treasurer, G. E. McEvers; Secretary, Lawrence Campbell. Meetings monthly.

